# High-quality EuO thin films the easy way via topotactic transformation

**DOI:** 10.1038/ncomms8716

**Published:** 2015-07-16

**Authors:** Thomas Mairoser, Julia A. Mundy, Alexander Melville, Daniel Hodash, Paul Cueva, Rainer Held, Artur Glavic, Jürgen Schubert, David A. Muller, Darrell G. Schlom, Andreas Schmehl

**Affiliations:** 1 Zentrum für Elektronische Korrelationen und Magnetismus, Universität Augsburg, Universitätsstraße 1, 86159 Augsburg, Germany.; 2 School of Applied and Engineering Physics, Cornell University, Ithaca, New York 14853, USA.; 3 Department of Materials Science and Engineering, Cornell University, Ithaca, New York 14853, USA.; 4 Quantum Condensed Matter Division, Oak Ridge National Laboratory, Oak Ridge, Tennessee 37831, USA.; 5 Peter Grünberg Institute, PGI 9-IT, JARA-FIT, Research Centre Jülich, 52425 Jülich, Germany.; 6 Kavli Institute at Cornell for Nanoscale Science, Ithaca, New York 14853, USA.

## Abstract

Epitaxy is widely employed to create highly oriented crystalline films. A less appreciated, but nonetheless powerful means of creating such films is via topotactic transformation, in which a chemical reaction transforms a single crystal of one phase into a single crystal of a different phase, which inherits its orientation from the original crystal. Topotactic reactions may be applied to epitactic films to substitute, add or remove ions to yield epitactic films of different phases. Here we exploit a topotactic reduction reaction to provide a non-ultra-high vacuum (UHV) means of growing highly oriented single crystalline thin films of the easily over-oxidized half-metallic semiconductor europium monoxide (EuO) with a perfection rivalling that of the best films of the same material grown by molecular-beam epitaxy or UHV pulsed-laser deposition. As the technique only requires high-vacuum deposition equipment, it has the potential to drastically improve the accessibility of high-quality single crystalline films of EuO as well as other difficult-to-synthesize compounds.

## Abstract (web summary)

In the absence of matching substrates, the growth of oxide thin films can be challenging. Here, the authors demonstrate the growth of EuO thin films via a topotactic reaction, where a chemical reaction transforms a single crystal of one phase into that of another.

## Graphical abstract

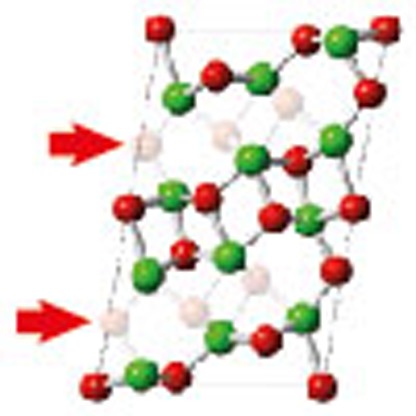

Europium metal can adopt three stable valence states, Eu^2+^ in europium monoxide (EuO, rock salt structure, space group *Fm*
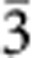
*m*) (ref. 1), Eu^2 2/3+^ in Eu_3_O_4_ (orthorhombic structure, space group *Pnma*)^2^ and Eu^3+^ in Eu_2_O_3_ (monoclinic or cubic structure, space groups *C*2/*m* or *Ia*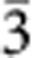, respectively)^3^,^4^. Of these phases, EuO is scientifically the most interesting compound, mainly because it is a rare example of a high-magnetization ferromagnetic semiconductor (7 *μ*_B_ per Eu; *T*_C_=69 K) (ref. 5) which can be epitactically integrated with silicon^6^, GaAs^7^, GaN^6^, graphene^8^, diamond^9^ and many oxide substrates. It is a half metal with over 96% spin polarization and can be conductance matched to silicon by rare-earth doping, rendering it an ideal candidate for spintronic applications^6^,^10^. In addition, it shows a plethora of giant transport and magneto-optic properties, which makes it a fascinating material for basic research as well as device applications^6^.

As EuO has its highest potential when epitactically integrated with spintronic-relevant semiconductors, the requirements with respect to the structural perfection of EuO films are basically the same as for all other semiconductor applications. Decades of technological innovation make it possible to fabricate silicon and other mainstream semiconductors with unrivalled perfection. For thin films, the standard methods all involve epitaxy—utilizing liquids (liquid-phase epitaxy), gases (vapour-phase epitaxy) or solids (solid-phase epitaxy). In this process, the atoms in the film inherit their crystalline arrangement from the underlying single crystal on which they are deposited and with which they do not react. If the depositing materials react with the substrate, the result is often disastrous to the crystalline perfection of the product formed, but an exception exists: topotaxy. Here the product inherits its highly oriented structure from the single crystal it replaced^11^.

Although rare, topotactic transformations can occur as materials oxidize (for example, Fe to FeO, Cu to Cu_2_O) (ref. 12), alter their hydrogen content (for example, FeO(OH) to Fe_2_O_3_) (ref. 11), swap anions (for example, ZnS to ZnO) (ref. 13) or undergo phase transitions^14^ (for example, the α to β transition in quartz)^11^. They are known for many mineral systems (for example, the formation of spinel from sapphire and periclase)^11^,^15^ and have even been observed in meteorites^16^. Most involve the substitution or addition of ions to a structure, but examples involving reduction are known^17^,^18^,^19^,^20^,^21^,^22^ and have been used to create epitactic films of phases that are otherwise inaccessible including LaNiO_2_, SrFeO_2_, CaFeO_2_ and even superlattices of these topotactically transformed phases^23^,^24^,^25^,^26^. This approach is being exploited in thin films due to its experimental simplicity^27^, for example, the formation of SrFeO_3-α_F_γ_ from epitactic SrFeO_3-*x*_ films, where the corrosive fluorine can be introduced *ex situ* after the epitactic film growth is complete^28^.

In many metal oxides the desired physical properties depend strongly on the structure of the compound, which often can be delicately controlled by altering the oxygen stoichiometry and thereby the oxidation state or valence of the involved metals. Controlling the oxygen stoichiometry is therefore of great importance in the preparation of oxides in the form of epitactic thin films for basic research or applications. For many materials, the desired phase is the fully oxidized one and its synthesis merely requires a sufficiently oxidizing environment during deposition. This makes their growth comparatively easy, for example, by reactive sputtering, molecular-beam epitaxy (MBE) or pulsed-laser deposition (PLD) in a (relatively) high-pressure ambient of oxygen or even ozone. For the case of EuO, however, the phase with the lowest oxidation state shows by far the most interesting physical properties. To access this phase one faces much higher demands, mainly the need to provide a controlled environment that is neither too oxidative nor too reducing during both growth and cooling following growth.

In this Article we demonstrate an alternative approach that does not require ultra-high-vacuum (UHV) equipment to grow high-quality EuO films, but can be executed in standard high-vacuum deposition systems, which are much more abundant. This technique allows the growth of single crystalline EuO films by magnetron sputtering under high-vacuum conditions with crystalline quality surpassing that of the best films grown by UHV techniques. The growth mechanism is based on a chemical reduction of an epitactic Eu_2_O_3_ precursor film into single crystalline EuO by chemical reduction. As the resulting high-quality EuO film inherits its crystalline structure form the precursor film, the reduction reaction is a topotactic transformation.

## Results

### Stability requirements and growth kinetics

At room temperature Eu^2+^ is only thermodynamically stable in minuscule oxygen partial pressures (below 10^–64^ atm)^29^, making its growth as well as subsequent *ex situ* processing highly demanding^6^. This instability in ambient conditions has, up to now, limited the growth of epitactic EuO films with high crystalline quality to UHV MBE^6^,^7^,^8^,^9^,^10^,^30^,^31^ and UHV PLD^32^,^33^. Sputtering of EuO films from an EuO target^34^,^35^ or multiple targets (Eu metal and Eu_2_O_3_) (refs 36, 37) has been reported, but resulted in polycrystalline, multiphase films, even when grown in a UHV deposition system.

For multivalent metals, thermodynamically relevant pressure values can be derived from Ellingham diagrams. According to thermodynamics, at 700 K, europium will only be stable in its lowest oxidation state (Eu^2+^ in EuO) if the oxygen partial pressure lies between 5 × 10^–80^ and 2 × 10^–57^ atm^29^,^38^. For thin-film growth, the kinetics of oxidation are also important^39^,^40^,^41^. To form EuO from incident molecular beams of europium and oxygen, the partial pressure of oxygen must be precisely calibrated so that the ratio of the sticking coefficients of europium atoms and O_2_ molecules imping on the film surface per unit time is 2:1. Deviations from this ratio lead to defects such as oxygen vacancies or higher Eu-oxides such as Eu_3_O_4_ and Eu_2_O_3_, unless self-limiting processes can be utilized. Because of this difficulty, EuO thin films are typically grown with adsorption-controlled techniques^30^ requiring well-controlled oxygen partial pressures in the UHV range and limiting deposition temperatures to values above ∼400 °C. Also, epitactic growth of many other multivalent metals requires sophisticated and expensive deposition equipment such as UHV MBE or PLD systems effectively limiting the access to high-quality epitactic films of such materials.

### Four-circle X-ray diffraction (XRD) structural analysis

**Figure 1:**
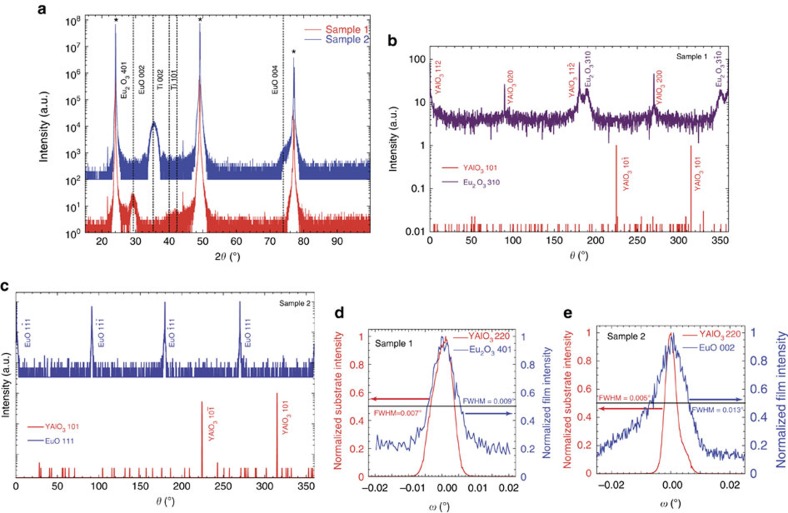
Establishing the orientation relationship of the topotactic transformation. (**a**) *θ*–2*θ*-scans of a single-phase (401)-oriented Eu_2_O_3_ film (sample 1) and a single-phase (001)-oriented EuO film capped with titanium (sample 2) grown on (110) YAlO_3_. Thickness fringes are present around the EuO 002 peak. Substrate peaks are marked by asterisks. (**b**) *φ*-scans of the YAlO_3_ 101 and the monoclinic Eu_2_O_3_ 310 families of peaks. These scans demonstrate the epitactic growth of the Eu_2_O_3_ film with in-plane orientation relationship 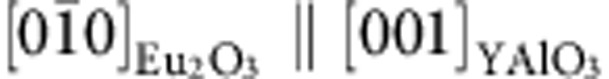 (scan parameters: 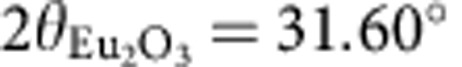; 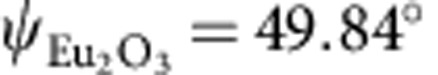; 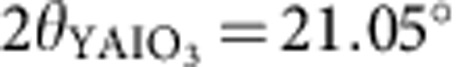; 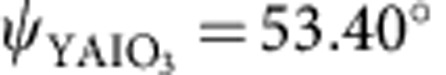). (**c**) *φ*-scans of the YAlO_3_ 101 and the EuO 111 families of peaks. The four EuO peaks demonstrate the topotactic conversion of Eu_2_O_3_ into an untwinned (001)-oriented EuO film on the (110) YAlO_3_ substrate with in-plane orientation relationships 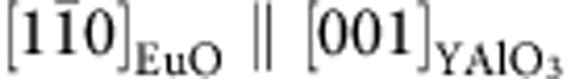 (scan parameters: 2*θ*_EuO_=30.08°; *ψ*_EuO_=52.50°; 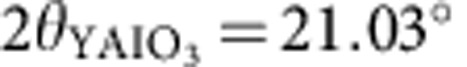; 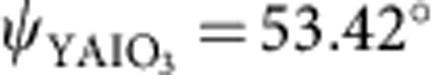). (**d**) Rocking curves of the YAlO_3_ 220 peak and the Eu_2_O_3_ 401 peak, measured on sample 1, respectively. The FWHM of the film peak is comparable to that of the substrate peak, indicating the excellent crystalline quality. (**e**) Rocking curves of the YAlO_3_ 220 peak and the EuO 002 peak, measured on sample 2, respectively. The FWHM of the film peak is only slightly larger than that of the film peak of sample 1.

The crystalline quality of all films was investigated using four-circle XRD. To demonstrate the transformation reaction, two Eu_2_O_3_ films were deposited simultaneously on (110) YAlO_3_ (ref. 42). Sample 1 was then taken out of the sputter system, while (after air exposure) sample 2 was heated back up to *T*_D_≈580 °C and capped with titanium as described in Methods. Figure 1a shows the *θ*–2*θ*-scans of both samples. Sample 1 corresponds to (401)-oriented single-phase monoclinic Eu_2_O_3_. The *θ*–2*θ*-scan of sample 2, however, corresponds to a (001)-oriented single-phase EuO film. The orientation relationships between the films and substrates were determined by off-axis XRD scans. Figure 1b shows *φ*-scans of the YAlO_3_ substrate and the Eu_2_O_3_ film of sample 1. In combination with calculated orientation relationships (Supplementary Figs 1 and 2 and Supplementary Table 1), the data shows epitactic growth with an in-plane orientation relationship 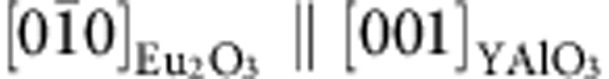. The *φ*-scans of sample 2 (Fig. 1c) correspond to single crystalline EuO with an in-plane orientation relationship to the substrate of 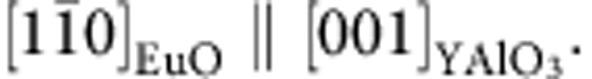 Rocking curves of the Eu_2_O_3_ 401 peak and the EuO 002 peak (Fig. 1d,e) demonstrate the outstanding crystalline quality of the Eu_2_O_3_ and the EuO films, respectively. The full width at half maximum (FWHM) of the Eu_2_O_3_ 401 peak of 0.009° is comparable to that of the substrate (0.007°). The FWHM of 0.013° of the EuO 002 peak of sample 2 is only slightly larger (Fig. 1e). For samples where the titanium layer was deposited *in situ*, directly after the Eu_2_O_3_ deposition, a FWHM of the EuO 002 peak as small as 0.004° has been observed using a substrate with a comparably small FWHM. This is the smallest value reported in the literature, smaller even than the best films grown by MBE or UHV PLD^30^,^33^. Using X-ray reflectometry, a root-mean-squared (r.m.s.) interface roughness of 0.28 nm between the EuO film and the TiO_*x*_ layer was measured. This value is smaller than the r.m.s. roughness of the substrate surface measured by X-ray reflectometry of 0.70 nm, indicating a high-quality interface (Supplementary Fig. 4).

As the XRD data demonstrate, it is the hot capping of the Eu_2_O_3_ with titanium that leads to a reduction of the precursor films to single crystalline, topotactic, high-quality EuO films. During this reaction the titanium metal is oxidized to TiO_*x*_. This reduction induced by the titanium capping is a topotactic transformation, a chemical reaction that results in a material with a crystalline orientation related to the starting product^11^,^43^,^44^.

### STEM and EELS analysis

**Figure 2:**
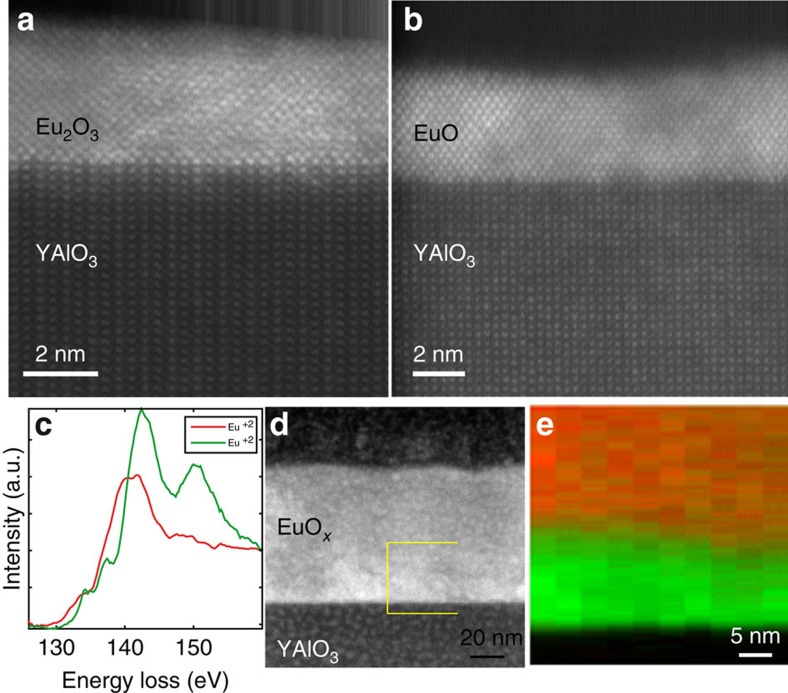
Characterization of the europium valence and film microstructure. (**a**) Cross-sectional HAADF-STEM image of the Eu_2_O_3_ film (sample 1). (**b**) Cross-sectional HAADF-STEM image of the transformed EuO film (sample 2) viewed along the [110] zone axis of the EuO film and the 
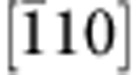
 zone axis of the YAlO_3_. See Supplementary Fig. 3 for a lower magnification STEM image of this same sample showing a larger area. (**c**) EELS fine structure analysis of the EuO M_4.5_ edge. Distinct EELS fingerprints corresponding to the occurrence of only Eu^3+^ in sample 1 and of only Eu^2+^ in sample 2, respectively. (**d**) Cross-sectional HAADF-STEM image of a partially reduced EuO film. The EELS analysis was performed on the region indicated by the yellow box. A non-negative nonlinear least squares fit to the components in **c** reveals the distribution of the EuO (Eu^2+^ is shown in red) and Eu_2_O_3_ (Eu^3+^ is shown in green) in the partially reduced film as shown in (**e**). Streaks at the top of the STEM images are an artifact of post-acquisition drift correction.

To verify this transformation mechanism and to characterize the film microstructure, europium valence and interface abruptness, the same samples shown in Fig. 1 were investigated by scanning transmission electron microscopy (STEM) and electron energy-loss spectroscopy (EELS). Figure 2a shows the high-angle annular dark field (HAADF) signal from the Eu_2_O_3_ film (sample 1) and Fig. 2b shows the HAADF signal from the EuO film (sample 2). The Eu_2_O_3_ film is epitactic to the substrate and exhibits distinct Eu^3+^ valence revealed by EELS. The Eu^2+^ and Eu^3+^ Eu–N_4,5_ edges are shown in Fig. 2c^45^,^46^. The transformed EuO film, sample 2, also formed a coherent interface with the YAlO_3_ substrate. A diffractogram from the film (Fig. 1a) shows a pattern consistent with the EuO rock salt crystal structure. The EELS signal depicted in Fig. 2c shows a distinct fingerprint, consistent with the Eu^2+^ state^45^,^46^. Lateral scans across the film did not yield any variations in the europium valence as would be expected for a film with predominately EuO and potentially small remnant inclusions of Eu_2_O_3_ (or Eu_3_O_4_). Therefore, we conclude that the film is phase pure and does not contain any remnant inclusions of Eu_2_O_3_ or Eu_3_O_4_ within the detection limit of EELS.

The transformation of the Eu_2_O_3_ precursor film to EuO is driven by chemical reduction with titanium being the reducing agent. The reduction of Eu^3+^ to Eu^2+^ is described by the chemical reaction: Eu_2_O_3_+Ti→2 EuO+TiO with ΔG° of −461 and −423 kJ mol^−1^ at 300 and 900 K, respectively^29^,^38^. The formation of the monoxide of titanium (TiO) is energetically favoured (Supplementary Table 2). From these free energies of reaction and the ideal gas constant *R*, the equilibrium constant


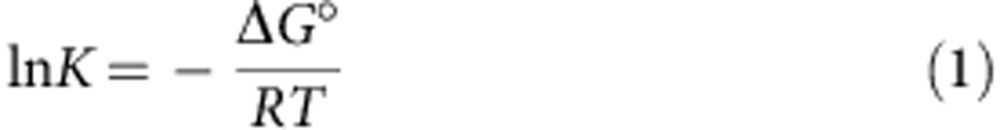


of this reaction can be calculated to be *K*_300 K_=5.9 × 10^80^ and *K*_900 K_=1.6 × 10^74^. The equilibrium constant is much larger than one, indicating the almost complete conversion of Eu^3+^ into Eu^2+^. Note that the formation of europium metal is thermodynamically disallowed because of the positive value of ΔG° for all corresponding formation reactions (Supplementary Table 2). Presumably the same reason precludes the formation of Eu_3_O_4_, though we are not aware of free energy data for Eu_3_O_4_.

As the reduction of Eu^3+^ to Eu^2+^ requires oxygen diffusion from the precursor film to the titanium capping layer, the transformation propagates from the titanium layer into the Eu_2_O_3_ film. Figure 2d shows a HAADF-STEM image and Fig. 2e, the valence analysis of the EELS signal of an only partially reduced film. The EELS signal was fitted with a linear least square optimization to the europium reference spectra shown in Fig. 2c to determine the spatial variance in the europium valence. Close to the titanium layer, only Eu^2+^ is present, whereas near the substrate, only Eu^3+^ can be found. The depth of the reduction strongly depends on the oxygen diffusion length and hence on the substrate temperature during the titanium deposition. We find that at room temperature <1 nm of the Eu_2_O_3_ is reduced to EuO, whereas at 600 °C at least 60 nm of EuO can be transformed. We note that the partially oxidized titanium capping layer protects the EuO film from re-oxidation when exposed to air. This allows *ex situ* measurements of the films without the need for further protection.

### Magnetic properties

**Figure 3:**
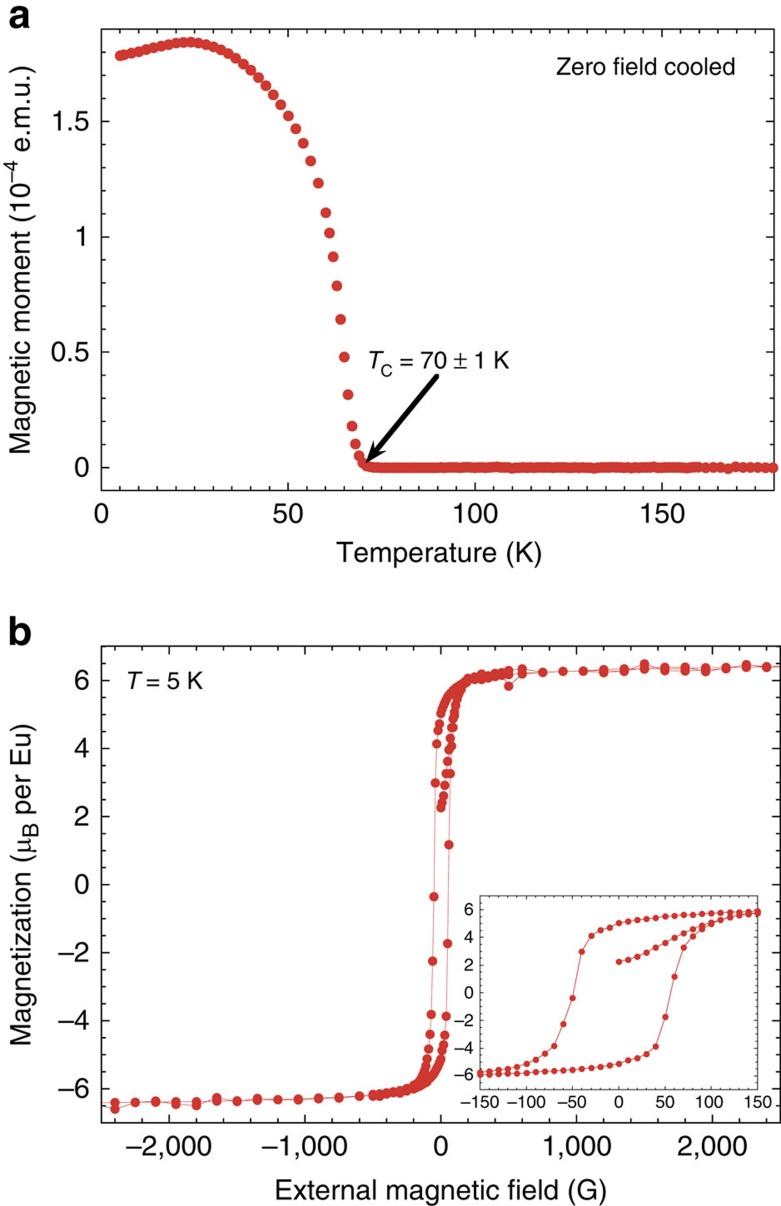
Magnetic properties of topotactic EuO. (**a**) Spontaneous magnetization *M*(*T*) of an EuO film grown on (110) YAlO_3_ via a topotactic transformation, measured using superconducting quantum interference device magnetometry in zero external field. Both the shape of the *M*(*T*) curve and the extracted *T*_C_ is typical of EuO films. The decrease of *M*(*T*) at low temperatures is due to domain effects. (**b**) *M*(*μ*_0_*H*) curve of the same sample. The saturation magnetization of the film was determined to be 
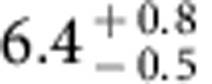
 *μ*_B_ per Eu. The inset shows a magnified view of the hysteresis curve, which has a coercive field of 50±10 G.

The high crystalline quality results in excellent physical properties of the transformed EuO films. The Curie temperature (*T*_C_) and the magnetic field dependence of the magnetization (*M*(*T*) and *M*(*μ*_0_*H*)) of the films were determined using superconducting quantum interference device magnetometry. Figure 3a shows a typical zero-field *M*(*T*) measurement from which *T*_C_=70±1 K was determined^47^. This value equals that of bulk EuO (ref. 48). Figure 3b shows the in-plane *M*(*μ*_0_*H*) characteristic at *T*=5 K of the same film. The saturation magnetization of 
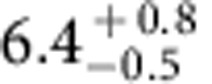
 *μ*_B_ per Eu is in good agreement with the expected saturation magnetization of 7 *μ*_B_ per Eu originating from the 4*f* (ref. 7) electronic configuration of Eu^2+^ (ref. 5). The very low coercive field of 50±10 G is again consistent with the excellent crystalline quality of the film.

## Discussion

**Figure 4:**
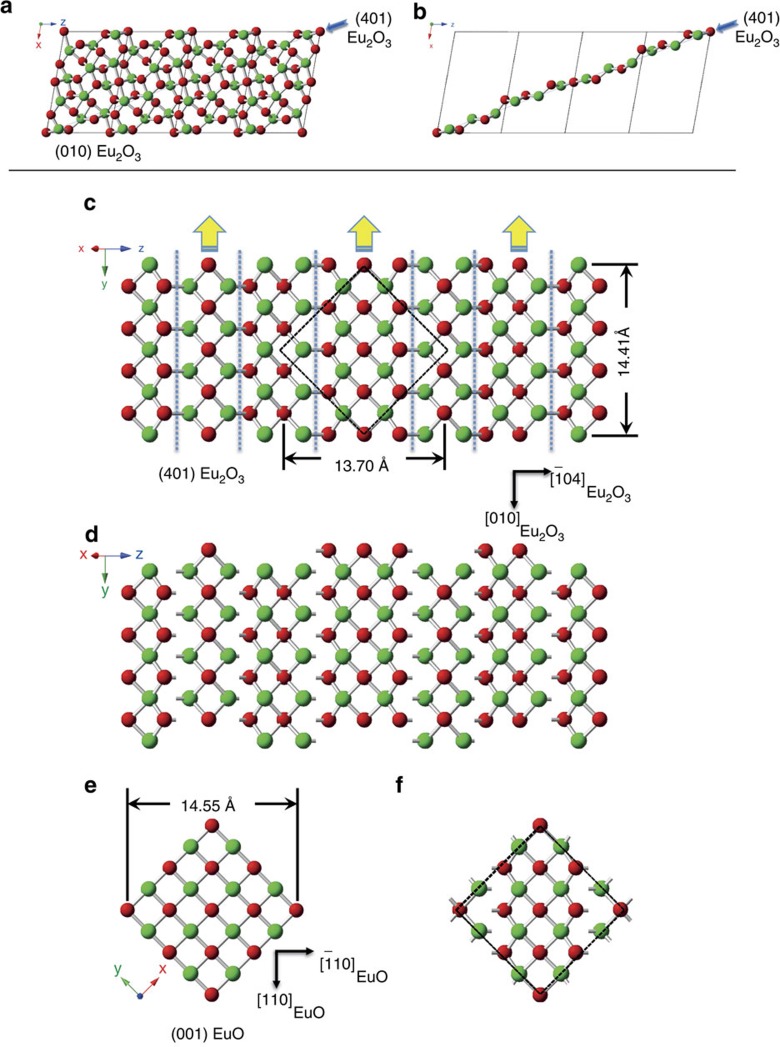
A comparison of the atomic arrangements of the (401) Eu_2_O_3_ and (001) EuO planes. (**a**) The crystal structure of the monoclinic polymorph of Eu_2_O_3_ viewed along the [010] zone axis. The unit cell of Eu_2_O_3_ is outlined with dashed lines and the (401) plane, which is viewed edge-on, is indicated by the blue line. (**b**) The atoms closest to the chosen (401) plane (the blue line) have overall composition EuO and form a closely packed layer. (**c**) The (401) plane of atoms selected in **b** viewed at normal incidence. The atomic arrangement is similar to that of the (001) EuO plane and can be made virtually identical by shifting the columns of atoms between the dashed lines in the direction of the yellow arrows by 
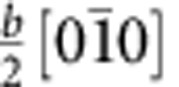
. (**d**) The (401) plane of Eu_2_O_3_ after performing the atomic shifts described in **c**. (**e**) The (001) EuO plane. (**f**) Emphasizes the similarity in atomic arrangement between **d** and **e**, by showing the overlap unit cell of shifted 
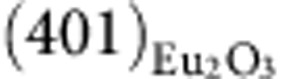
. The overlap unit cell between 
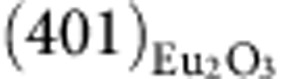
 and (001)_EuO_ is also shown by the dashed quadrilateral (a rhombus for 
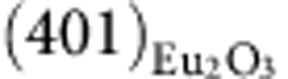
 and a square for (001)_EuO_) in **c**, before any shift of the atomic positions.

From the orientation relationship between the initial epitactic Eu_2_O_3_ film and the (110) YAlO_3_ substrate on which it was grown (Supplementary Fig. 1) and the orientation relationship of the topotactically transformed EuO film with this same substrate (Supplementary Fig. 2), we know the alignment between the reactant and product phases: 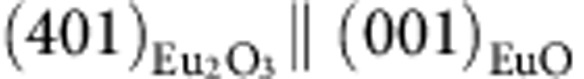 with 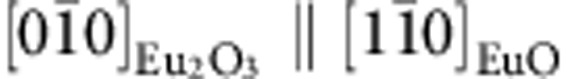. This orientation relationship is shown in Fig. 4. Motifs of structural similarity and a reasonable lattice match (–1.0% along 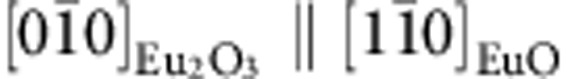 and –5.8% along 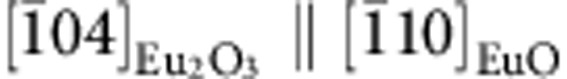) are evident between the (401) Eu_2_O_3_ and (001) EuO planes. Interestingly, the shortest distance between europium atoms in the Eu_2_O_3_ before the topotactic transformation (the 
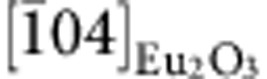
 direction) ends up being nearly parallel to the shortest distance between europium atoms in the EuO following the transformation (the 
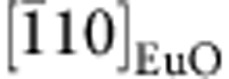
 direction). This same behaviour has also been noted in other topotactic transformations: Fe to FeO (ref. 49) and Ba to BaO (ref. 50). A plausible pathway for the topotactic transformation between Eu_2_O_3_ and EuO is shown in Fig. 4. The closely packed (401) plane of Eu_2_O_3_ in Fig. 4b,c has composition EuO. Shifting the indicated columns of atoms in the (401) plane of Eu_2_O_3_ by 1.8 Å along 
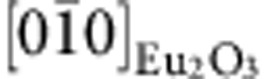
 (see Fig. 4c) results in virtually identical atomic patterns in the (401) Eu_2_O_3_ and (001) EuO planes.

In conclusion, we have demonstrated the topotactic transformation of epitactic Eu_2_O_3_ films grown on (110) YAlO_3_ into high-quality, single crystalline EuO films by a titanium-induced chemical reduction. The approach only requires high-vacuum deposition equipment, overcoming the prior need for UHV deposition systems to grow films of comparable quality. STEM, EELS and superconducting quantum interference device measurements corroborate the growth mechanism and the excellent quality of the transformed EuO films. The method used is general; provided the structures of the precursor and the target films are closely related, the transformations are expected to be topotactic leading to high-quality single crystalline films.

## Methods

### Film growth

All films were grown in a high-vacuum sputter deposition system (base pressure ≈3 × 10^−7^ mbar) using 3′′ targets (99.99% purity). In the course of this study, the sputtering system was equipped with a more powerful vacuum pump, reducing the base pressure to ≈3 × 10^−8^ mbar with no detectable changes in film quality. The films were grown on single crystalline YAlO_3_ substrates oriented within 0.2° of (110). (110) oriented YAlO_3_ provides a rectangular surface net with in-plane lattice constants 7.431 Å along 
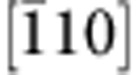
 and 7.370 Å along [001] at room temperature^42^. EuO is cubic with lattice constant 5.144 Å at room temperature^1^. The result is a lattice mismatch of 2.1% for 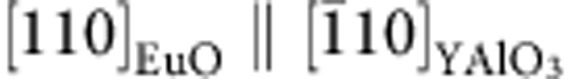 and 1.3% for 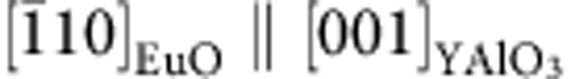 (ref. 6). The substrates were glued to a resistive heater using silver paint. The substrate heater was then installed in the deposition system, which was evacuated to its base pressure. The substrates were then heated in vacuum to their deposition temperature (*T*_D_≈580 °C). During substrate heating the chamber background pressure usually increased to about 5 × 10^−6^ mbar. Eu_2_O_3_ was deposited for 20 min by reactive radio-frequency magnetron sputtering at a power of *P*_rf_=50 W using an Ar/O_2_ working gas mixture (total pressure *p*_tot_=0.05 mbar). To provide constant growth conditions during the deposition, both working gases were constantly renewed at flow rates of 2.0 sccm. After the Eu_2_O_3_ deposition, the films were capped with titanium by d.c. magnetron sputtering (deposition time=4 min, *P*_d.c._=100 W, argon working gas, *P*_tot_=0.05 mbar, argon flow=4.0 sccm). Subsequently the heater was switched off and the films were cooled to room temperature in vacuum.

## Additional information

**How to cite this article:** Mairoser, T. *et al*. High-quality EuO thin films the easy way via topotactic transformation. *Nat. Commun.* 6:7716 doi: 10.1038/ncomms8716 (2015).

## Supplementary Material

Supplementary Information Supplementary Figures 1-4, Supplementary Tables 1-2 and Supplementary References

## References

[b1] Eick H. A., Baenziger N. C. & Eyring L. Lower oxides of samarium and europium. The preparation and crystal structure of SmO_0.4-0.6_, SmO and EuO. J. Am. Chem. Soc. 78, 5147–5149 (1956) .

[b2] Rau R. C. The crystal structure of Eu_3_O_4_. Acta Crystallogr. 20, 716–723 (1966) .

[b3] Yakel H. L. A refinement of the crystal structure of monoclinic europium sesquioxide. Acta Crystallogr. B 35, 564–569 (1979) .

[b4] Bommer H. Z. Die Gitterkonstanten der C-Formen der Oxyde der seltenen Erdmetalle. Z. Anorg. Allg. Chem. 241, 273–280 (1939) .

[b5] Matthias B. T., Bozorth R. M. & Van Vleck J. H. Ferromagnetic interaction in EuO. Phys. Rev. Lett. 7, 160–161 (1961) .

[b6] Schmehl A. . Epitaxial integration of the highly spin-polarized ferromagnetic semiconductor EuO with silicon and GaN. Nat. Mater. 6, 882–887 (2007) . 17873862 10.1038/nmat2012

[b7] Swartz A. G. . Epitaxial EuO thin films on GaAs. Appl. Phys. Lett. 97, 112509 (2010) .

[b8] Swartz A. G., Odenthal P. M., Hao Y., Ruoff R. S. & Kawakami R. K. Integration of the ferromagnetic insulator EuO onto graphene. ACS Nano 6, 10063 (2013) . 23083411 10.1021/nn303771f

[b9] Melville A. . Epitaxial growth of europium monoxide on diamond. Appl. Phys. Lett. 103, 22402 (2013) .

[b10] Melville A. . Lutetium-doped EuO films grown by molecular-beam epitaxy. Appl. Phys. Lett. 100, 222101 (2012) .

[b11] Shannon R. D. & Rossi R. C. Definition of topotaxy. Nature 202, 1000–1001 (1964) .

[b12] Mehl R. F., McCandless E. L. & Rhines F. N. Orientation of oxide films on metals. Nature 134, 1009 (1934) .

[b13] Yamaguti T. Oxidation of a crystal surface studied by means of cathode ray reflection. Proc. Phys. Math. Soc. Jpn 17, 443–453 (1935) .

[b14] Katz G., Nicol A. W. & Roy R. New topotaxy in precipitation from spinel. Nature 223, 609–610 (1969) .

[b15] Rossi R. C. & Fulrath R. M. Epitaxial growth of spinel by reaction in the solid state. J. Am. Ceram. Soc. 46, 145–149 (1963) .

[b16] Tomioka N. & Fujino K. Natural (Mg,Fe)SiO_3_-ilmenite and -perovskite in the Tenham Meteorite. Science 277, 1084–1086 (1997) . 9262473 10.1126/science.277.5329.1084

[b17] Mehl R. F. & McCandless E. L. Orientation of oxide films on iron. Nature 137, 702 (1936) .

[b18] Cahn R. W. Solid-state chemistry: topotaxy in metal-oxide reduction. Nature 316, 297 (1985) .

[b19] Revcolevschi A. & Dhalenne G. Crystallographically aligned metal-oxide composite made by reduction of a directionally solidified oxide-oxide eutectic. Nature 316, 335–336 (1985) .

[b20] Hayward M. A., Green M. A., Rosseinsky M. J. & Sloan J. Sodium hydride as a powerful reducing agent for topotactic oxide deintercalation: synthesis and characterization of the nickel(I) oxide LaNiO_2_. J. Am. Chem. Soc. 121, 8843–8854 (1999) .

[b21] Tsujimoto Y. . Infinite-layer iron oxide with a square-planar coordination. Nature 450, 1062–1065 (2007) . 18075589 10.1038/nature06382

[b22] Hadermann J., Abakumov A. M., Adkin J. J. & Hayward M. A. Topotactic reduction as a route to new close-packed anion deficient perovskites: structure and magnetism of 4H-BaMnO_2+*x*_. J. Am. Chem. Soc. 131, 10598–10604 (2009) . 19722633 10.1021/ja903216d

[b23] Inoue S. . Single-crystal epitaxial thin films of SrFeO_2_ with FeO_2_ ‘infinite layers'. Appl. Phys. Lett. 92, 161911 (2008) .

[b24] Kawai M. . Reversible changes of epitaxial thin films from perovskite LaNiO_3_ to infinite-layer structure LaNiO_2_. Appl. Phys. Lett. 94, 082102 (2009) .

[b25] Inoue S. . Anisotropic oxygen diffusion at low temperature in perovskite-structure iron oxides. Nat. Chem. 2, 213–217 (2010) . 21124479 10.1038/nchem.547

[b26] Matsumoto K. . Artificial superlattice thin film of infinite-layer structure [CaFeO_2_]/[SrFeO_2_]. Appl. Phys. Express 3, 105601 (2010) .

[b27] Zurbuchen M. A. Topotactic anion exchange oxide films and method of producing the same. US Patent 7,208,044 B2 (2007) .

[b28] Moon E. J. . Fluorination of epitaxial oxides: synthesis of perovskite oxyfluoride thin films. J. Am. Chem. Soc. 136, 2224–2227 (2014) . 24443775 10.1021/ja410954z

[b29] Pankratz L. B. Thermodynamic Properties of Elements and Oxides United States Bureau of Mines (1982) .

[b30] Ulbricht R. W., Schmehl A., Heeg T., Schubert J. & Schlom D. G. Adsorption-controlled growth of EuO by molecular-beam epitaxy. Appl. Phys. Lett. 93, 102105 (2008) .

[b31] Sutarto R. . Epitaxial and layer-by-layer growth of EuO thin films on yttria-stabilized cubic zirconia (001) using MBE distillation. Phys. Rev. B 79, 205318 (2009) .

[b32] Beukers J. . Epitaxial EuO thin films by pulsed laser deposition monitored by *in situ* X-ray photoelectron spectroscopy. Thin Solid Films 518, 5173–5176 (2010) .

[b33] Yamasaki T., Ueno K., Tsukazaki A., Fukumura T. & Kawasaki M. Observation of anomalous Hall effect in EuO epitaxial thin films grown by a pulse laser deposition. Appl. Phys. Lett. 98, 082116 (2011) .

[b34] Lee K. & Suits J. C. Reduction of ferromagnetic exchange with lattice dilation in EuO. J. Appl. Phys. 41, 954–956 (1970) .

[b35] Coburn J. W. & Lee K. Sputter deposition of EuO thin films. J. Appl. Phys. 42, 5903–5905 (1971) .

[b36] Barbagallo M. . Experimental and theoretical analysis of magnetic moment enhancement in oxygen-deficient EuO. Phys. Rev. B 81, 235216 (2010) .

[b37] Barbagallo M. . Thickness-dependent magnetic properties of oxygen-deficient EuO. Phys. Rev. B 84, 075219 (2011) .

[b38] Barin I. & Platzki G. Thermochemical Data of Pure Substances VCH (1995) .

[b39] Kubinski D. J., Hoffman D. W. & Soltis R. E. Reactive codeposition of *in situ* Y-Ba-Cu-O superconducting films using dilute mixtures of ozone in oxygen. J. Appl. Phys. 71, 1860–1867 (1992) .

[b40] Matijasevic V. C. . MBE synthesis of YBa_2_Cu_3_O_y_ superconducting thin films. IEEE Trans. Appl. Supercond. 3, 1524–1527 (1993) .

[b41] Appelboom H. M. . Sm-Ba-Cu-O films grown at low temperature and pressure. Physica C 214, 323–334 (1993) .

[b42] Geller S. & Wood E. A. Crystallographic studies of perovskite-like compounds. I. Rare earth orthoferrites and YFeO_3_, YCrO_3_, YAlO_3_. Acta Crystallogr. 9, 563–568 (1965) .

[b43] Oswald H. R. Chemische Anwendungen der Elektronenmikroskopie: Topotaktische Reaktionen. Angew. Chem. 81, 470 (1969) .

[b44] Bailey S. W. . Report of the International Mineralogical Association (IMA)—International Union of Crystallography (IUCr) Joint Committee of Nomenclature. Acta Crystallogr. A 33, 681–684 (1977) .

[b45] Mundy J. A. . Hetero-epitaxial EuO interfaces studied by analytic electron microscopy. Appl. Phys. Lett. 104, 091601 (2014) .

[b46] Negusse E. . Effect of electrode and EuO thickness on EuO-electrode interface in tunneling spin filter. J. Appl. Phys. 99, 08E507 (2006) .

[b47] Mairoser T., Loder F., Melville A., Schlom D. G. & Schmehl A. Influence of chemical doping on the magnetic properties of EuO. Phys. Rev. B 87, 014416 (2013) .

[b48] McGuire T. R. & Shafer M. W. Ferromagnetic europium compounds. J. Appl. Phys. 35, 984–988 (1964) .

[b49] Mehl R. F. & McCandless E. L. Oxide films on iron. Trans. Am. Inst. Mining Met. Eng. 125, 531–559 (1937) .

[b50] Burgers W. G. & van Amstel J. J. A. P. ‘Oriented' oxidation of barium. Physica 3, 1057–1063 (1936) .

