# Supplementary material for: High-quality EuO thin films the easy way via topotactic transformation
Source: Nat Commun. 2015 Jul 16;6:7716. doi: 10.1038/ncomms8716 (PMC4518251; doi:10.1038/ncomms8716)
Supplement: Supplementary Information — Supplementary Figures 1-4, Supplementary Tables 1-2 and Supplementary References [file ncomms8716-s1.pdf]

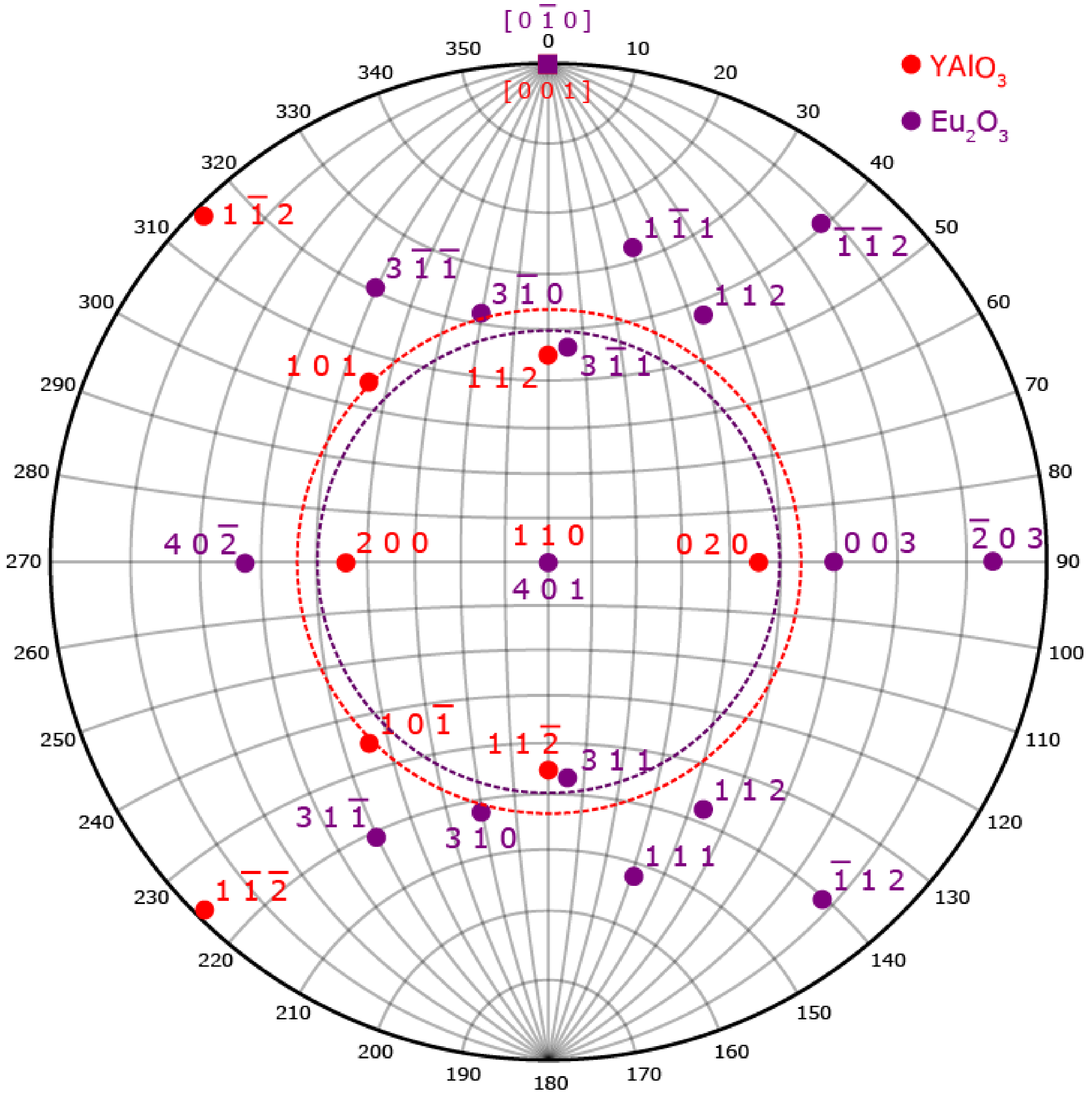

Supplementary Figure 1: Stereographic projection of the relevant poles of single crystals of monoclinic Eu_2O_3 (purple) [1] and $YAlO_3$ (red) [2]. Bulk lattice constants of these phases are used. These poles are superimposed to show the observed epitactic orientation relationship deduced for our epitactic Eu_2O_3 films grown on $YAlO_3$ substrates: (401) Eu_2O_3 || (110) $YAlO_3$ with $[0\bar{1}0]_{Eu_2O_3} \parallel [001]_{YAlO_3}$. All poles in the northern hemisphere of the stereographic projection (relevant for the reflection diffraction geometry used) of monoclinic Eu_2O_3 and $YAlO_3$ that lie within ±4 degrees in 2θ of the 2θ angle used for the ϕ-scan of Fig. 1b ($2\theta = 31.6°$) are shown. The dashed circles indicate the trajectories of the ϕ-scans in Fig. 1b. The discrepancy between the calculated ψ angle of the 310 and $3\bar{1}0$ peaks of bulk monoclinic Eu_2O_3 ($\psi = 54.8°$) and the value at which the peaks in the ϕ-scan were found to occur ($\psi = 49.8°$) is attributed to a distortion of the unit cell of the monoclinic Eu_2O_3 film.

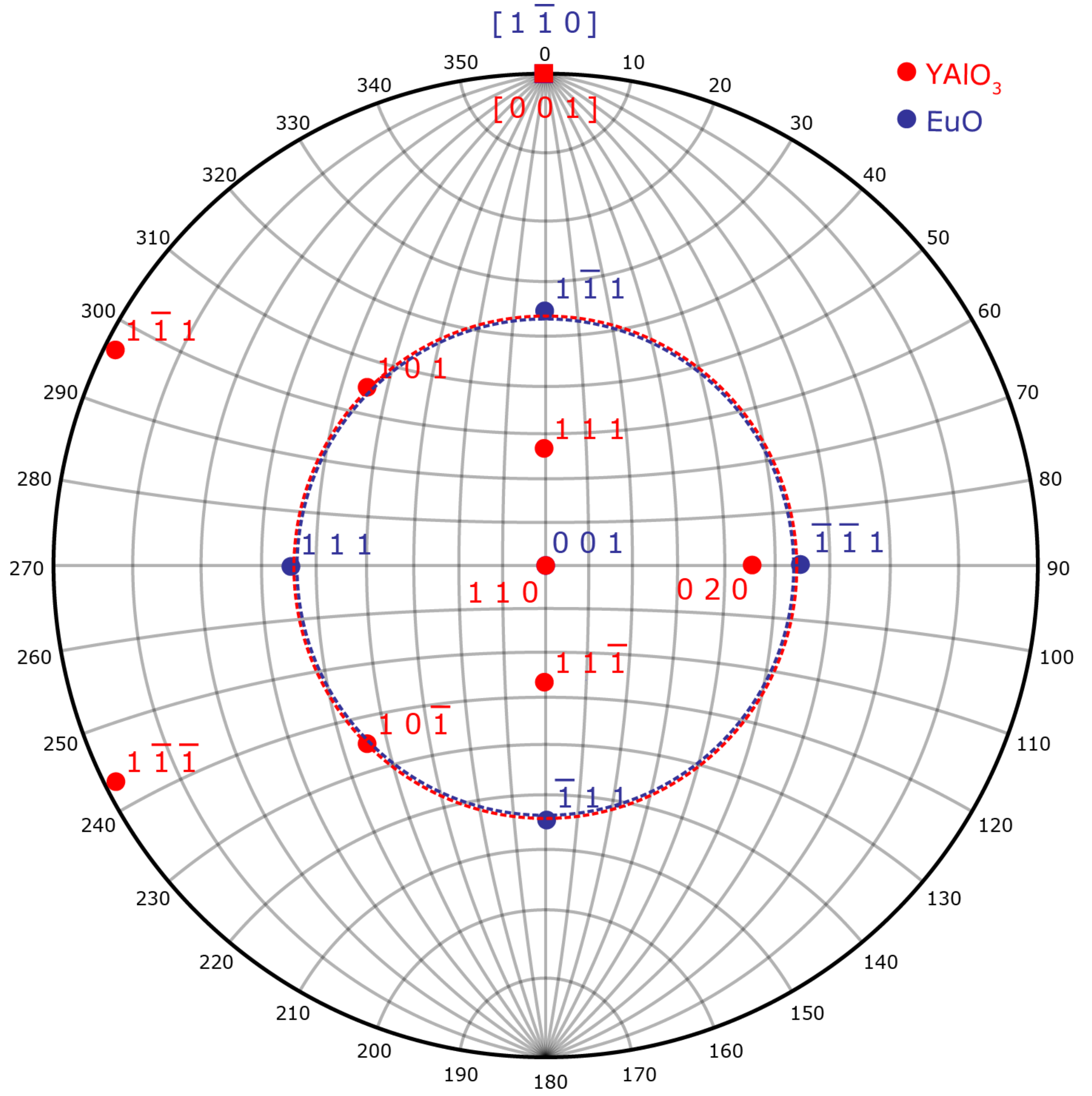

Supplementary Figure 2: Stereographic projection of the relevant poles of single crystals of EuO (blue) [3] and $YAlO_3$ (red) [2]. Bulk lattice constants of these phases are used. These poles are superimposed to show the observed epitactic orientation relationship deduced for our topotactically transformed EuO films grown on $YAlO_3$ substrates: (001) EuO || (110) $YAlO_3$ with [1$\bar{1}$0] EuO || [001] $YAlO_3$. All poles in the northern hemisphere of the stereographic projection (relevant for the reflection diffraction geometry used) of EuO and $YAlO_3$ that lie within ±4 degrees in 2θ of the 2θ angle used for the ϕ-scan of Fig. 1c ($2\theta = 30.1°$) are shown. The dashed circles indicate the trajectories of the ϕ-scans in Fig. 1c.

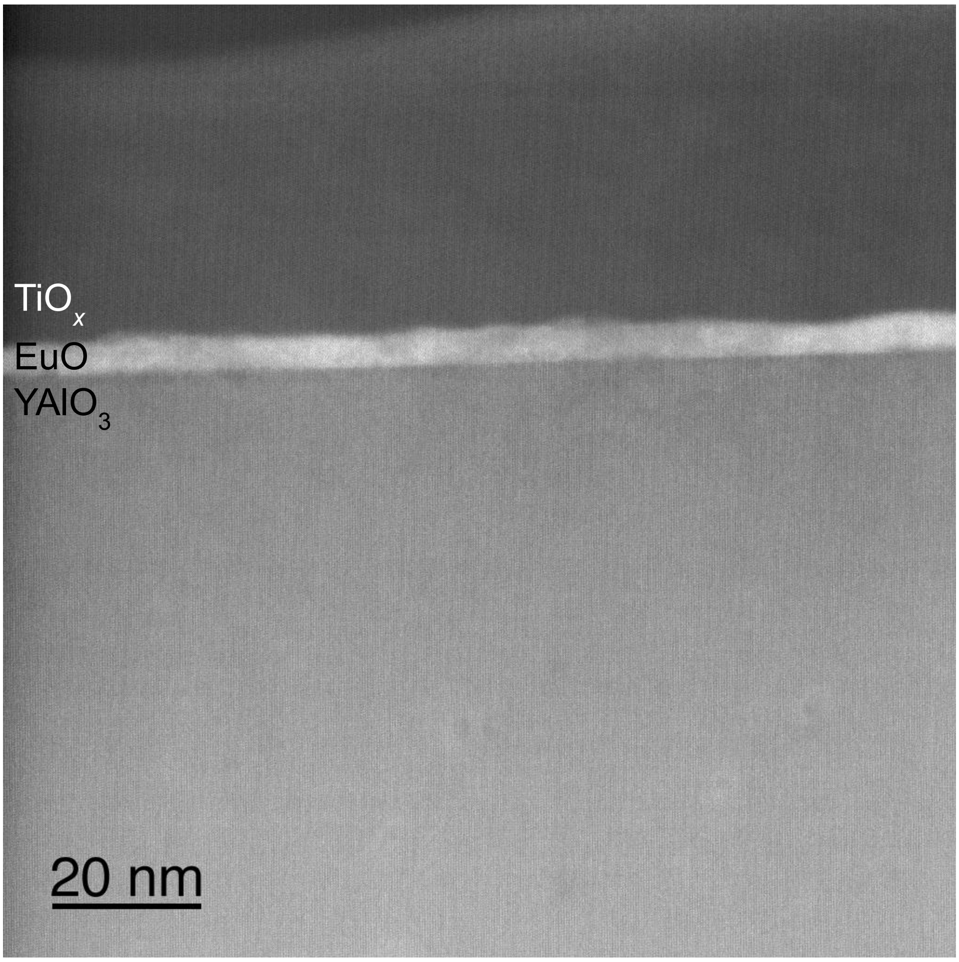

Supplementary Figure 3: Low magnification HAADF-STEM image of the same EuO film and TiO_x capping layer imaged with high-resolution in Fig. 2b.

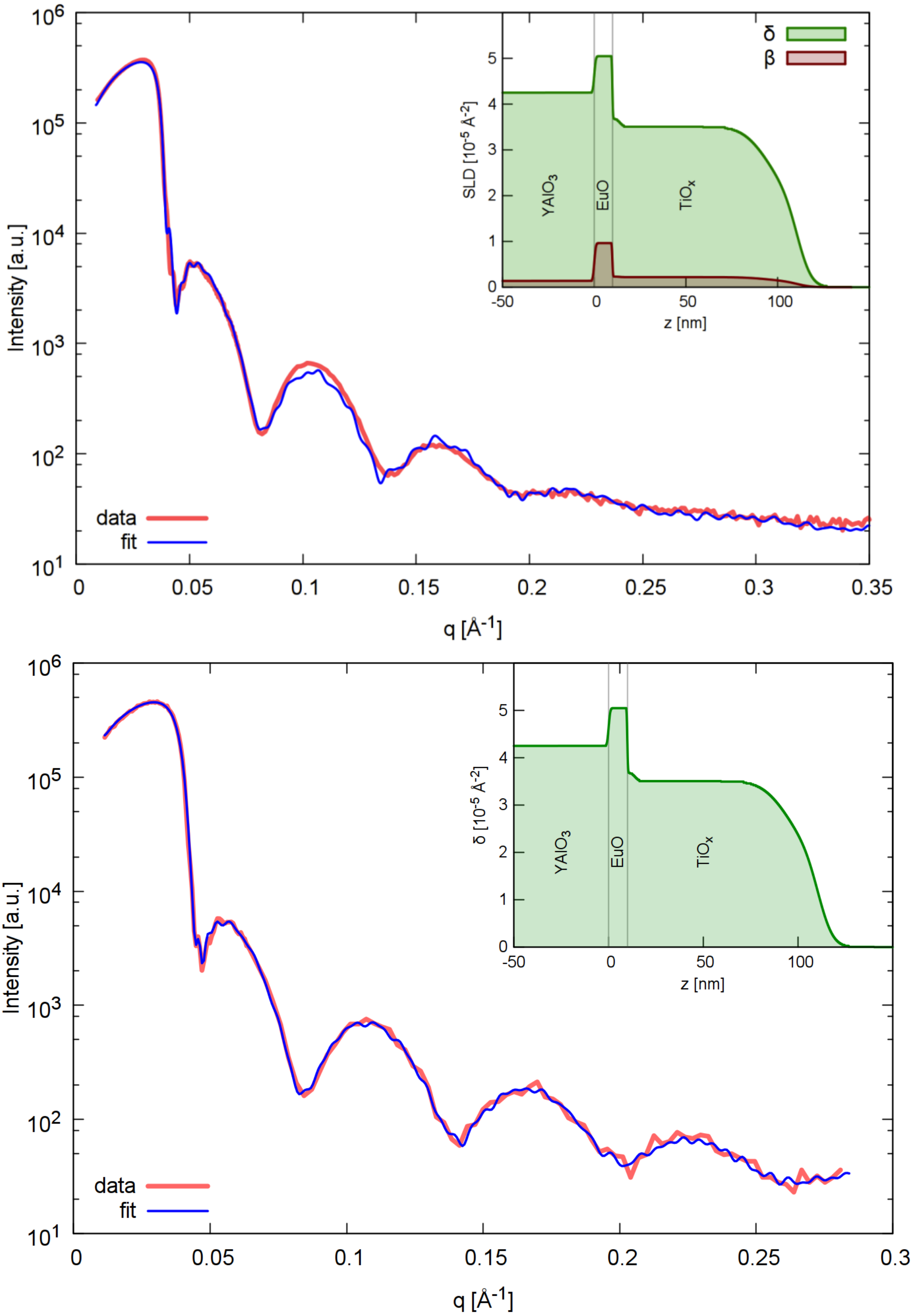

Supplementary Figure 4: X-ray reflectometry measurement used to determine both the film thickness and the interface roughness of a topotactically transformed EuO film. The figure shows the measured (red) and simulated (blue) x-ray reflectivity of a Ti/TiO_x/EuO/$YAlO_3$ sample. The derived film thicknesses were then used as input parameter to calculate the saturation magnetization of the films. The best-fit simulation corresponds to a film thickness of 9.86 nm, an rms interface roughness between the substrate and the EuO film of 0.70 nm, and an EuO/TiO_x rms interface roughness of 0.28 nm. The inset shows the real (δ) and imaginary parts (-β) of the scattering length densities. The high contrast between Ti and Eu supports the high sensitivity of the measurement.

Crystal	Structure	Space group	Lattice parameter	Reference
$YAlO_3$	orthorhombic	*Pbnm*	a = 5.179 Å	[1]
			b = 5.329 Å	
			c = 7.370 Å	
EuO	rock salt	$Fm\bar{3}m$	a = 5.144 Å	[3]
Eu_2O_3	Monoclinic	$C2/m$	a = 14.11 Å	[2]
			b = 3.602 Å	
			c = 8.808 Å	
			β = 100.037°	

Supplementary Table 1: Bulk lattice parameters of $YAlO_3$, EuO, and Eu_2O_3 used to calculate the pole figures of Figs. S1 and S2.

Reaction	ΔG^0 at 300 K (kJ/mol)	ΔG^0 at 900K (kJ/mol)
$Eu_2O_3 + Ti \rightarrow 2\ EuO + TiO$	-461	-423
$2\ Eu_2O_3 + Ti \rightarrow 4\ EuO + TiO_2$	-33	-66
$3\ Eu_2O_3 + 2\ Ti \rightarrow 6\ EuO + Ti_2O_3$	-149	-196
$5\ Eu_2O_3 + 3\ Ti \rightarrow 10\ EuO + Ti_3O_5$	-176	-268
$7\ Eu_2O_3 + 4\ Ti \rightarrow 14\ EuO + Ti_4O_7$	-216	-340
$Eu_2O_3 + 3\ Ti \rightarrow 2\ Eu + 3\ TiO$	26	17
$2\ Eu_2O_3 + 3\ Ti \rightarrow 4\ Eu + 3\ TiO_2$	464	427
$Eu_2O_3 + 2\ Ti \rightarrow 2\ Eu + Ti_2O_3$	132	117
$5\ Eu_2O_3 + 9\ Ti \rightarrow 10\ Eu + 3\ Ti_3O_5$	880	761
$7\ Eu_2O_3 + 12\ Ti \rightarrow 14\ Eu + 3\ Ti_4O_7$	1325	1172
$EuO + Ti \rightarrow Eu + TiO$	57	58

Supplementary Table 2: Calculated Gibbs free energies (ΔG^0) of possible reactions between Eu_2O_3 and titanium at 300 K and 900 K. These temperatures encompass the range of growth temperatures utilized in this work. All possible reactions involving constituents for which the free energies are tabulated in refs. [4] and [5] are listed. This table of reactions indicates that the formation of EuO and TiO_x is thermodynamically allowed whereas the formation of europium metal and TiO_x cannot occur.

Supplementary references

[1] Yakel, H. L. A refinement of the crystal structure of monoclinic europium sesquioxide. *Acta Crystallogr., Sect. B: Struct. Crystallogr. Cryst. Chem.* **35**, 564–569 (1979).

[2] Geller, S. & Wood, E.A. Crystallographic studies of perovskite-like compounds. I. Rare earth orthoferrites and $YFeO_3$, $YCrO_3$, $YAlO_3$. *Acta Crystallogr.* **9**, 563–568 (1965).

[3] Eick, H. A., Baenziger, N. C. & Eyring, L. Lower oxides of samarium and europium. The preparation and crystal structure of $SmO_{0.4\text{-}0.6}$, SmO and EuO. *J. Am. Chem. Soc*. **78**, 5147–5149 (1956).

[4] Pankratz, L. B. *Thermodynamic Properties of Elements and Oxides* (United States. Bureau of Mines, Washington, D.C., 1982).

[5] Barin, I. & Platzki, G. *Thermochemical Data of Pure Substances* (VCH, Weinheim, 1995).
